# Suppression of inflammation by low-dose methotrexate is mediated by adenosine A_2A _receptor but not A_3 _receptor activation in thioglycollate-induced peritonitis

**DOI:** 10.1186/ar1914

**Published:** 2006-03-06

**Authors:** M Carmen Montesinos, Avani Desai, Bruce N Cronstein

**Affiliations:** 1 Department of Pharmacology, Universidad de Valencia, Burjassot, Valencia, Spain; 2 Department of Medicine, New York University School of Medicine, New York, USA

## Abstract

Prior studies demonstrate that adenosine, acting at one or more of its receptors, mediates the anti-inflammatory effects of methotrexate in animal models of both acute and chronic inflammation. Both adenosine A_2A _and A_3 _receptors contribute to the anti-inflammatory effects of methotrexate treatment in the air pouch model of inflammation, and the regulation of inflammation by these two receptors differs at the cellular level. Because different factors may regulate inflammation at different sites we examined the effect of low-dose weekly methotrexate treatment (0.75 mg/kg/week) in a model of acute peritoneal inflammation in adenosine A_2A _receptor knockout mice and A_3 _receptor knockout mice and their wild-type littermates. Following intraperitoneal injection of thioglycollate there was no significant difference in the number or type of leukocytes, tumor necrosis factor alpha (TNF-α) and IL-10 levels that accumulated in the thioglycollate-induced peritoneal exudates in adenosine A_2A _knockout mice or wild-type control mice. In contrast, there were more leukocytes, TNF-α and IL-10 in the exudates of the adenosine A_3 _receptor-deficient mice. Low-dose, weekly methotrexate treatment increased the adenosine concentration in the peritoneal exudates of all mice studied, and reduced the leukocyte accumulation in the wild-type mice and A_3 _receptor knockout mice but not in the A_2A _receptor knockout mice. Methotrexate reduced exudate levels of TNF-α in the wild-type mice and A_3 _receptor knockout mice but not the A_2A _receptor knockout mice. More strikingly, IL-10, a critical regulator of peritoneal inflammation, was increased in the methotrexate-treated wild-type mice and A_3 _knockout mice but decreased in the A_2A _knockout mice. Dexamethasone, an agent that suppresses inflammation by a different mechanism, was similarly effective in wild-type mice, A_2A _mice and A_3 _knockout mice. These findings provide further evidence that adenosine is a potent regulator of inflammation that mediates the anti-inflammatory effects of methotrexate. Moreover, these data provide strong evidence that the anti-inflammatory effects of methotrexate and adenosine are mediated by different receptors in different inflammatory loci, an observation that may explain why inflammatory diseases of some organs but not of other organs respond to methotrexate therapy.

## Introduction

Low-dose weekly methotrexate has become the mainstay treatment of rheumatoid arthritis and psoriasis, and it is the gold standard by which other systemic medications are measured in both disorders [1,2]. Methotrexate has been used to treat other inflammatory diseases including ankylosing spondylitis, multiple sclerosis and inflammatory bowel disease, but its efficacy in the therapy of these conditions is far less impressive [3-7].

An increasing body of evidence indicates that adenosine mediates, at least in part, the anti-inflammatory effects of methotrexate [8-13]. All known adenosine cell surface receptors (A_1_, A_2A_, A_2B _and A_3_) contribute to the modulation of inflammation, as demonstrated by many *in vitro *and *in vivo *pharmacologic studies (reviewed in [14,15]). We have previously demonstrated pharmacologically, using nonselective antagonists, that the anti-inflammatory effect of methotrexate is mediated by more than one subtype of adenosine receptor in the adjuvant arthritis model in the rat [16], and, using mice rendered deficient in A_2A _or A_3 _adenosine receptors, we found that both receptor subtypes are critical for the anti-inflammatory effects of methotrexate in the murine air pouch model of inflammation [17]. Since inflammation at different loci may be regulated by different cellular mechanisms, we determined whether the A_2A _and A_3 _receptors played similar roles in regulating inflammation in the peritoneum.

We examined the pharmacologic mechanism by which methotrexate diminishes inflammation in the thioglycollate-induced peritoneal inflammation model of acute inflammation in the mouse. We report here that, similar to the air pouch, methotrexate treatment increases peritoneal exudate adenosine concentrations in wild-type mice, A_2A _receptor knockout mice and A_3 _receptor knockout mice but, in contrast to the air pouch model, diminishes leukocyte accumulation only in the peritoneal exudates of A_3 _receptor knockout and wild-type mice, not of A_2A _knockout mice. Similarly, methotrexate decreased exudate tumor necrosis factor alpha (TNF-α) levels and increased IL-10 levels in wild-type mice and A_3 _knockout mice, but only marginally decreased TNF-α levels and significantly decreased IL-10 levels in A_2A _knockout mice.

## Materials and methods

### Materials

Thioglycollate medium (FTG) was obtained from Sigma Chemical Co. (St Louis, MO, USA). Methotrexate was purchased from Immunex (San Juan, PR, USA). All other materials were the highest quality that could be obtained.

### Animals

Mice with a targeted disruption of the gene for the adenosine A_2A _and A_3 _receptor have been described in detail elsewhere [18,19]. The mice used in these experiments were derived from four original heterozygous breeding pairs for each mouse strain. Mice described as wild type were specific for the related receptor knockout mice, since their background was different. Confirmation of mouse genotype was performed by PCR as previously described [17]. Mice were housed in the New York University animal facility, fed regular mouse chow and given access to drinking water *ad libitum*. All procedures described in the following were reviewed and approved by the Institutional Animal Care and Use Committee of New York University Medical Center and were carried out under the supervision of the facility veterinary staff.

### Peritoneal inflammation

Animals were given weekly intraperitoneal injections of either methotrexate (0.75 mg/kg, freshly reconstituted lyophilized powder) or vehicle (0.9% saline) for 4 weeks and the experiments were carried out within 3 days of the final dose of methotrexate. Dexamethasone (1.5 mg/kg) was administered by intraperitoneal injection 1 hour prior to induction of inflammation in the peritoneum. Thioglycollate peritonitis was induced by intraperitoneal injection of 0.5 ml sterile solution of thioglycollate medium (10% w/v in PBS) [20]. After 4 hours the animals were sacrificed by CO_2 _narcosis and their peritoneal cavities were lavaged with 3 ml cold PBS. The peritoneal area was massaged before withdrawing the lavage fluid. Exudates were maintained at 4°C until aliquots were diluted 1:1 with methylene blue (0.01% w/v in PBS) and cells were counted in a standard hemocytometer chamber. The concentration of adenosine and TNF-α in inflammatory exudates was quantified by HPLC and ELISA, respectively [17]. The IL-10 concentration in cell-free inflammatory exudates was quantified by ELISA (R&D Systems, Minneapolis, MN, USA) following the manufacturer's instructions.

### Statistical analysis

All statistical analyses were performed by SigmaStat software (SPSS, Inc., Chicago, IL, USA). Differences between groups were analyzed by one-way analysis of variance.

## Results

Since previous studies carried out in our laboratory showed that adenosine receptors play a pivotal role in the formation of the granulation tissue lining the air pouch [21], in a manner that might alter the inflammatory response, we sought to further evaluate the role of adenosine receptors in methotrexate-mediated suppression of inflammation in tissue that had not previously undergone injury or disruption. We therefore determined whether methotrexate inhibits acute leukocyte accumulation in thioglycollate-induced peritoneal inflammation in wild-type mice, adenosine A_2A _receptor knockout mice and adenosine A_3 _receptor knockout mice. Similar numbers of leukocytes accumulated in peritoneal inflammatory exudates of A_2A _knockout mice and their corresponding wild-type controls (Table 1). In contrast, there was a significant increase (20%) in the number of leukocytes that accumulated in peritoneal exudates of A_3 _knockout mice as compared with the wild-type controls (Table 1).

**Figure 1:**
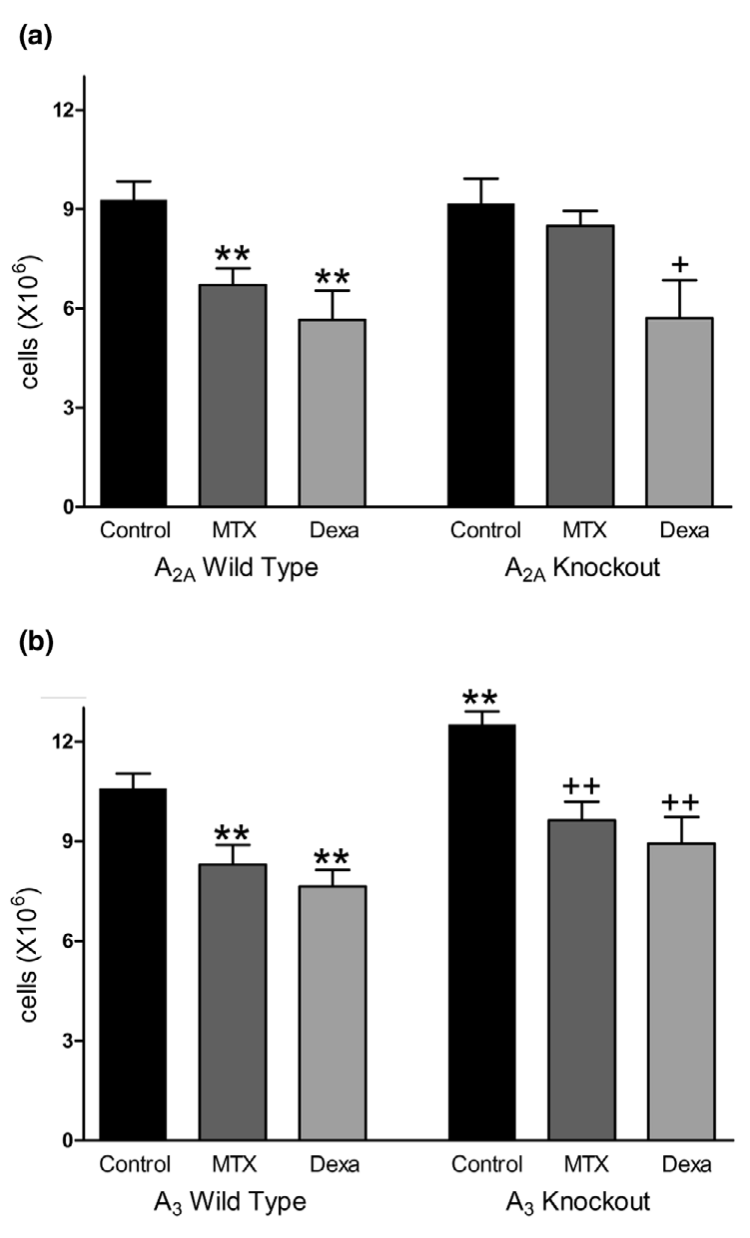
Effect of methotrexate and dexamethasone treatment on leukocyte accumulation in peritoneal exudates of mice. **(a) **A_2A _wild-type mice and A_2A _receptor knockout mice or **(b) **A_3 _wild-type mice and A_3 _receptor knockout mice either were treated with weekly injections of methotrexate (0.75 mg/kg) or saline control for 4 weeks prior to induction of inflammation or were treated with a single intraperitoneal injection of dexamethasone (1.5 mg/kg) or saline 1 hour before induction of inflammation and subsequent collection of inflammatory exudates, as described. Results are presented as the mean (± SEM) million cells per exudate. **P < 0.001 vs wild-type control mice, ^++^*P *< 0.001 vs knockout control mice, ^+^*P *< 0.05 vs knockout control mice, all one-way analysis of variance (Bonferroni *t *test).

Treatment with methotrexate increased the exudate adenosine concentration in wild-type mice, A_2A _knockout mice and A_3 _knockout mice (Table 2) and reduced the leukocyte accumulation in A_2A _wild-type mice by 30 ± 5% (*P *< 0.01 vs control, *n *= 7; Figure 1a), but reduced the leukocyte accumulation in the A_2A _knockout mice by only 7 ± 5% (*P *= not significant vs wild-type control, *n *= 6; Figure 1a). In contrast to the A_2A _knockout mice, methotrexate was no less effective as an anti-inflammatory agent in A_3 _receptor knockout mice (23 ± 5% inhibition, *P *< 0.001 vs A_3 _knockout control, *n *= 12; Figure 1b) than in A_3 _wild-type mice (22 ± 5% inhibition, *P *< 0.001 vs A_3 _wild-type control, *n *= 10; Figure 1b).

To determine whether the diminished anti-inflammatory effect of methotrexate in the A_2A _knockout mice was specific, we tested the effect of the potent steroidal anti-inflammatory agent dexamethasone in this model. Dexamethasone diminished leukocyte accumulation similarly in A_2A _wild-type mice, A_2A _knockout mice, A_3 _wild-type mice and A_3 _knockout mice (39 ± 9%, 38 ± 13%, 35 ± 4% and 36 ± 4% inhibition, *P *< 0.005, *P *< 0.05, *P *< 0.001 and *P *< 0.001 vs control, *n *= 4, *n *= 3, *n *= 9 and *n *= 9, respectively; Figure 1). Under the conditions studied there was no difference in the type of white cells that accumulated in the peritoneal cavities of either treated or untreated wild-type mice or knockout mice (>90% polymorphonuclear leukocytes).

In general, TNF-α accumulation in peritoneal exudates was much lower than previously reported in other models of inflammation, including carrageenan-induced inflammation in the air pouch and zymosan-induced peritoneal inflammation [17,22]. Similar to leukocyte accumulation, we found comparable levels of the proinflammatory cytokine TNF-α in peritoneal exudates of wild-type mice and A_2A _knockout mice, but significantly increased accumulation of TNF-α in peritoneal exudates of A_3 _knockout mice (Table 3). Methotrexate nevertheless inhibited TNF-α accumulation in peritoneal exudates of wild-type mice and A_3 _knockout mice more markedly than leukocyte accumulation (by 67% and 59%, respectively), and had a modest effect on TNF-α accumulation in peritoneal exudates of A_2A _knockout mice (Table 3). These findings are consistent with the prior observation that both A_2A _and A_3 _receptors modulate TNF-α production [23].

The cytokine IL-10, released by resident peritoneal macrophages, plays a regulatory anti-inflammatory role in the recruitment of leukocytes in murine models of peritoneal inflammation [22,24]. Since adenosine receptor activation modulates the release of IL-10 by different inflammatory cells [25-27] and methotrexate-treated rheumatoid arthritis patients have shown increased serum levels of this cytokine [28,29], we determined whether constitutively or methotrexate-modified IL-10 accumulation in the inflammatory exudate was altered in adenosine receptor-deficient mice. We found that, similar to the leukocyte infiltration and the TNF-α concentration, A_3 _knockout mice had significantly higher IL-10 levels in their peritoneal inflammatory exudates when compared with wild-type mice and A_2A _knockout mice (Table 4). As expected, treatment with methotrexate stimulated IL-10 accumulation in the exudate by 56% in wild-type mice, but significantly decreased IL-10 levels in exudates of A_2A_-deficient mice. Although methotrexate increased IL-10 levels in the exudates of methotrexate-treated A_3 _knockout mice, this increase did not achieve statistical significance. Due to the high variability in the IL-10 levels we found in our experiments, it would require between 30 and 60 mice per group to achieve statistical significance.

These results provide evidence that the anti-inflammatory effects of methotrexate (and adenosine) are mediated by different receptors in different loci. Specifically, in contrast to our previously published observation that both A_2A _and A_3 _receptors are required for the anti-inflammatory effects in the air pouch model of inflammation, only the A_2A _receptor is required to suppress inflammation in the peritoneal space.

## Discussion

The purine nucleoside adenosine is a ubiquitous autacoid present in all tissues and body fluids. Under basal conditions, the extracellular adenosine concentration is rather constant (30–300 nM), but its concentration can increase dramatically to 10 μM or even higher, as a result of ATP catabolism, when there is an imbalance between energy use and energy supply, such as in oxygen depletion, or when there is cell necrosis as a consequence of mechanical or inflammatory injury. Adenosine acts via four distinct adenosine receptor subtypes – the adenosine A_1_, A_2A_, A_2B_, and A_3 _receptors – that are all members of the large family of seven-transmembrane spanning, heterotrimeric G protein-associated receptors, coupling to classical second messenger pathways such as modulation of cAMP production or the phospholipase C pathway. In addition, they couple to mitogen-activated protein kinases, which could give them a role in cell growth, survival, death and differentiation (reviewed in [30]).

Adenosine is a potent endogenous anti-inflammatory agent, and all four adenosine receptor subtypes participate in this effect (reviewed in [14]). All cell subtypes involved in the inflammatory process differentially express functional adenosine receptors. It is well documented that microvascular endothelial cells, major players conducting the movement of leukocytes between tissue compartments, express adenosine A_2A _and A_2B _receptors [31,32]. Pharmacological and molecular approaches have shown that neutrophils, monocytes and macrophages express all four adenosine receptor subtypes. Although adenosine A_1 _receptor activation has been associated with proinflammatory properties in inflammatory cell types [33-35], the anti-inflammatory effect of selective A_1 _agonists acting in the central nervous system has been demonstrated *in vivo *[36-38]. Adenosine A_2A _receptor activation inhibits neutrophil and monocyte oxidative burst, degranulation and release of cytokines and chemokines [39-41]. Activation of A_2B _receptors selectively inhibits collagenase mRNA accumulation in synovial fibroblasts and mediates neutrophil-stimulated intestinal epithelial leakiness [42,43]. Adenosine A_3 _receptors have also been described as anti-inflammatory in human blood leukocytes and in murine models of inflammation [19,44-46].

The results of these reported studies confirm the anti-inflammatory effects of adenosine acting at A_3 _receptors because animals deficient in this receptor show an exacerbated response to the inflammatory insult. Moreover, we found that more polymorphonuclear leukocytes accumulate in the peritoneal exudates of A_3 _knockout mice in comparison with their wild-type littermates, consistent with the hypothesis that this receptor plays a greater role as an endogenous regulator of inflammation. Our data are in agreement with prior reports showing that adenosine A_3 _receptor agonists suppress the expression and production of macrophage inflammatory protein 1α, a chemokine that enhances neutrophil recruitment into inflammatory sites [45], and suppress the production of TNF-α by lipopolysaccharide-stimulated macrophages [19]. Adenosine A_3 _receptor agonists thus ameliorate joint inflammation in several murine models of arthritis [45,46].

Monocytes and macrophages synthesize and release into their environment a variety of cytokines and other proteins that play a central role in the development of acute and chronic inflammation. It has been firmly established that adenosine modulates the production of inflammatory cytokines, including TNF-α, IL-10, and IL-12 [23,25-27,47]. In addition to the regulatory effect of adenosine in cytokine secretion, we have further established that Th1 proinflammatory cytokine IL-1 and TNF-α treatment increases message and protein expression of A_2A _and A_2B _receptors by both microvascular endothelial cells and THP-1 monocytoid cells. IFN-γ treatment also increased the expression of A_2B _receptors, but decreased the expression of A_2A _receptors [25,32,48]. It is therefore probably at inflamed sites, where proinflammatory cytokines such as IL-1 and TNF-α are abundantly secreted, mostly by monocytes/macrophages, that the subsequent upregulation of A_2A _and A_2B _receptors on endothelial cells and other inflammatory cells along with endogenous adenosine release constitutes a feedback loop to suppress further inflammation. The demonstration that adenosine receptors expressed in microvascular endothelial cells are modified during inflammation suggests an important role for these receptors in the increased angiogenesis and vascular permeability that characterize both acute and chronic inflammatory responses. Moreover, in previous studies, activation of both A_2A _and A_2B _receptors on either endothelial cells or macrophages has been reported to enhance the expression of vascular endothelial growth factor and to promote angiogenesis [21,49-51].

Methotrexate is an effective disease-modifying drug widely used in low doses at weekly intervals for the control of rheumatoid arthritis and psoriasis with a relatively safe profile compared with other therapies [1,2]. Since folate administration prevents many of the toxicities of methotrexate without affecting the therapeutic effects [52], there is little support for the hypothesis that inhibition of folate-dependent pathways (for example, cellular proliferation) is responsible for the therapeutic effects of the agent. Following administration, methotrexate is taken up by cells and undergoes polyglutamation, resulting in the intracellular accumulation of the long-lived polyglutamates of methotrexate. These metabolites, in addition to inhibiting folate metabolism, directly inhibit 5-aminoimidazole-4-carboxamide ribonucleotide transformylase, resulting in an intracellular accumulation of 5-aminoimidazole-4-carboxamide ribonucleotide, which is an intermediate metabolite in the *de-novo *pathway of purine synthesis, and has been associated with increases in extracellular adenosine [9,13,53].

There is now increasing evidence that accumulation of adenosine at sites of inflammation plays a pivotal role in the anti-inflammatory effect of methotrexate. *In vitro *studies showed that methotrexate produces adenosine release by human fibroblasts and endothelial cells [53], and *in vivo *studies showed that methotrexate is ineffective in the presence of antagonists of adenosine or adenosine deaminase (the enzyme responsible for the deamination of adenosine to inosine) in animal models of acute and chronic inflammation [8]. Moreover, adenosine receptor antagonists and deletion of adenosine receptors eliminates the anti-inflammatory response to methotrexate in animal models of acute and chronic inflammation and patients with rheumatoid arthritis [13,16,54].

Although the contribution of adenosine to the mechanism of action of methotrexate is well accepted, it is still unclear which adenosine receptors participate in the effect of methotrexate. Results of early studies, using pharmacological tools, suggested that the adenosine A_2A _receptor was the main receptor subtype involved in suppressing inflammation [8]. In the model of adjuvant arthritis in rats, however, we found that only nonselective adenosine receptor antagonists could block the protective effect of methotrexate whereas selective antagonists of individual adenosine receptors did not alter the response to methotrexate [16], consistent with involvement of multiple adenosine receptors. Using knockout animals we observed that both A_2A _and A_3 _adenosine receptors are involved in methotrexate-mediated suppression of air pouch inflammation [17] but, as reported here, only A_2A _receptors are involved in methotrexate-mediated suppression of peritoneal inflammation. Methotrexate exerted similar anti-inflammatory effects in wild-type mice and A_3 _knockout mice, but failed to inhibit leukocyte and TNF-α accumulation in A_2A _knockout mice. Moreover, methotrexate treatment augmented the accumulation of IL-10, a known anti-inflammatory cytokine, in wild-type mice and A_3 _knockout mice, but actually decreased IL-10 levels in A_2A _knockout mice. We do not have a clear explanation for this other than to note it is probable that in the MTX-treated A_2A _knockout mice there is an imbalance in A_1 _adenosine receptor function in the absence of A_2A_, consistent with the previous observation of Hasko and colleagues that an A_1 _adenosine receptor agonist reduces IL-10 release by lipopolysaccharide-stimulated RAW macrophages [27]. IL-10 is therefore, as previously reported, a critical regulator of peritoneal inflammation that is regulated by A_2A _adenosine receptors but not by A_3 _adenosine receptors [24,25].

We infer from these results and previous reports that the involvement of different adenosine receptor subtypes depends upon the site of and stimulus for inflammation. We therefore conclude it is probable that the requirement for activation of multiple adenosine receptor subtypes in the pharmacologic control of chronic inflammation results from the involvement of different types of inflammatory cells and disease-specific differences in the inflammatory environment.

## Conclusion

The studies reported here provide strong evidence that adenosine mediates the anti-inflammatory effects of methotrexate at doses relevant to those used to treat inflammatory arthritis. These results indicate that agents which interact with adenosine A_2A _receptors directly or promote adenosine release at inflamed sites may be useful for the treatment of inflammatory conditions, whereas occupancy of other adenosine receptors may be involved in suppression of inflammation in a site-specific fashion.

## Abbreviations

ELISA = enzyme-linked immunosorbent assay; HPLC, high performance liquid chromatography; IL = interleukin; PBS, phosphate-buffered saline; PCR = polymerase chain reaction; TNF-α = tumor necrosis factor alpha.

## Competing interests

MCM and AD declare that they have no competing interests. BNC declares the following competing interests: consultant – King Pharmaceuticals, Tap Pharmaceuticals, Can-Fite Pharmaceuticals, Bristol-Myers Squibb, Regeneron, Centocor; grant support – NIH, King Pharmaceuticals; honoraria – Merck, Amgen; intellectual property – adenosine A_2A _receptors for wound healing, adenosine A_2A _receptor antagonists for fibrosis (both licensed to King Pharmaceuticals).

## Authors' contributions

MCM designed and coordinated the study, carried out the animal experimental procedures, performed the statistical analysis and drafted the manuscript. AD carried out the adenosine HPLC determinations and the immunoassays. BNC conceived of the study, participated in its design and corrected the manuscript. All authors read and approved the final manuscript.

## Figures and Tables

**Table 1:** Leukocyte accumulation in inflammatory exudates

Mouse group	Peritoneal exudate (× 10^6^cells ± SEM)
A_2A _wild type	9.3 ± 0.6 (*n *= 14)
A_2A _knockout	9.2 ± 0.8 (*n *= 14)
A_3 _wild type	10.6 ± 0.5 (*n *= 19)
A_3 _knockout	12.5 ± 0.4* (*n *= 23)

Inflammatory exudates were induced in the peritoneum of knockout and wild-type mice, as described. After 4 hours the exudates were collected and the leukocytes quantitated. The wild-type control mice were derived from the same heterozygous breeding pairs and were matched for age and sex. There was no difference in the number of leukocytes accumulating in the exudates of male vs female mice in either the knockout mice or wild-type mice. *P < 0.005 vs A_3 _wild-type mice, Student's *t *test.

**Table 2:** Adenosine concentration in peritoneal exudates

	Wild-type mice (nM ± SEM)	A_2A _knockout mice (nM ± SEM)	A_3 _knockout mice (nM ± SEM)
Control	118 ± 6 (*n *= 19)	110 ± 6 (*n *= 14)	133 ± 6 (*n *= 12)
Methotrexate (0.75 mg/kg/week)	178 ± 12* (*n *= 15)	162 ± 7** (*n *= 7)	214 ± 10^† ^(*n *= 9)

Wild-type mice, A_2A _receptor knockout mice or A_3 _receptor knockout mice were treated with either weekly injections of methotrexate (0.75 mg/kg) or saline control for 4 weeks prior to induction of inflammation. Inflammatory exudates were induced in the peritoneum of mice, as described. After 4 hours the exudates were collected and the adenosine levels quantitated. Wild-type data are a combination from both mouse strains. *P < 0.0001 vs wild-type control mice, Student's *t *test; **P < 0.0001 vs A_2A _knockout control mice, Student's *t *test; ^† ^*P *< 0.0001 vs A_3 _knockout control mice, Student's *t *test.

**Table 3:** Tumor necrosis factor alpha concentration in peritoneal exudates

	Wild-type mice (pg/ml ± SEM)	A_2A _knockout mice (pg/ml ± SEM)	A_3 _knockout mice (pg/ml ± SEM)
Control	42 ± 7 (*n *= 14)	36 ± 8 (*n *= 10)	75 ± 18* (*n *= 6)
Methotrexate (0.75 mg/kg/week)	14 ± 4** (*n *= 15)	25 ± 7 (*n *= 9)	31 ± 11^† ^(*n *= 8)

Wild-type mice, A_2A _receptor knockout mice or A_3 _receptor knockout mice were treated with either weekly injections of methotrexate (0.75 mg/kg) or saline control for 4 weeks prior to induction of inflammation. Inflammatory exudates were induced in the peritoneum of mice, as described. After 4 hours the exudates were collected, centrifuged at 100 × g and frozen. Tumor necrosis factor alpha levels were later quantitated by ELISA. Wild-type data are a combination from both mouse strains. **P < 0.001 vs wild-type control mice, Student's *t *test; *P < 0.05 vs wild-type control mice, Student's *t *test; ^†^*P *< 0.05 vs A_3 _knockout control mice, Student's *t *test.

**Table 4:** IL-10 concentration in peritoneal exudates

	Wild-type mice (pg/ml ± SEM)	A_2A _knockout mice (pg/ml ± SEM)	A_3 _knockout mice (pg/ml ± SEM)
Control	62 ± 7 (*n *= 24)	73 ± 9 (*n *= 12)	115 ± 14** (*n *= 15)
Methotrexate (0.75 mg/kg/week)	97 ± 18* (*n *= 12)	41 ± 6^† ^(*n *= 7)	150 ± 31 (*n *= 7)

Wild-type mice, A_2A _receptor knockout mice or A_3 _receptor knockout mice were treated with either weekly injections of methotrexate (0.75 mg/kg) or saline control for 4 weeks prior to induction of inflammation. Inflammatory exudates were induced in the peritoneum of mice, as described. After 4 hours the exudates were collected, centrifuged at 100 × g and frozen. IL-10 levels were later quantitated by ELISA. Wild-type data are a combination from both mouse strains. ** *P *< 0.001 vs wild-type control mice, Student's *t *test; * *P *< 0.05 vs wild-type control mice, Student's *t *test; ^†^*P *< 0.05 vs A_2A _knockout control mice, Student's *t *test.
